# Rare skin disease helps uncover mechanisms of acyl ceramide biosynthesis

**DOI:** 10.1016/j.jlr.2025.100916

**Published:** 2025-09-26

**Authors:** Susanne Tollinger, Daniela Ortner, Thomas Trafoier, Verena Moosbrugger-Martinz, Robert Gruber, Matthias Schmuth

**Affiliations:** 1 Department of Dermatology, Venereology and Allergy, Medical University of Innsbruck, Innsbruck, Austria; 2 Karl Landsteiner Institute for Pediatric Dermatology and Rare Diseases, Innsbruck, Austria; 3 Member of the European Reference Network for Rare Skin Diseases (ERN Skin)

**Keywords:** genetics, rare disease, skin barrier, lipids, ceramide

Omega-O-acyl ceramides in the epidermis are composed of three different carbon chains. A long chain base, most often sphingosine, is combined with an omega-hydroxylated ultra-long-chain fatty acid (28–36 carbon atoms) and with linoleic acid (Fig. 1A). Thus, as opposed to most ceramides, omega-O-acyl ceramides have three rather than two hydrophobic chains and contain ultra-long fatty acids, unique to the outer layers of the epidermis. The work of Schratter *et al*. (1) published in this issue of the *Journal of Lipid Research* illustrates how we can learn from genetic *ABHD5* (1-acylglycerol-3-phosphate O-acyltransferase) variants about the molecular and spatial requirements for omega-O-acyl ceramide synthesis mediating epidermal permeability barrier function.

**Fig. 1:**
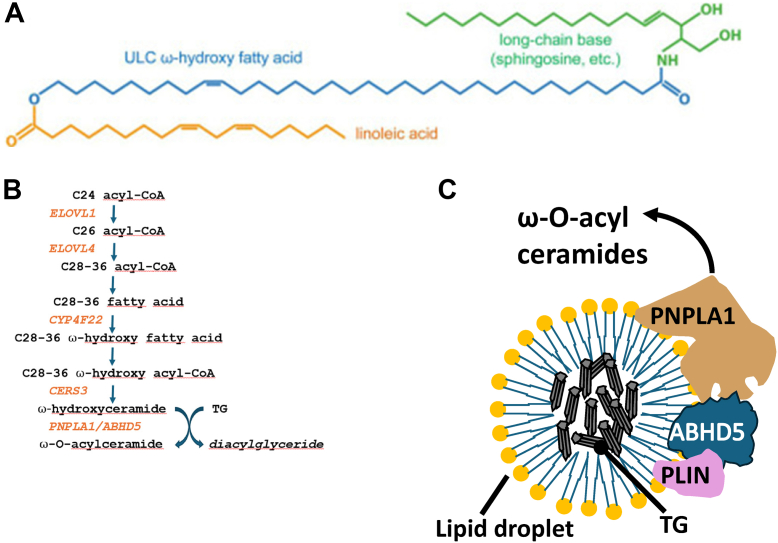
Omega-O-acyl ceramide structure, biosynthesis, and colocalization of enzymes and cofactors in proximity to lipid droplets. A: Structure of omega-O-acyl ceramide. Modified from the study by Kien *et al*. (2). B: Current knowledge of the biosynthesis pathway. C: Putative scheme of the proximity between *PNPLA1*, *ABHD5*, lipid droplets, and perilipin (PLIN). Modified from the study by Schratter *et al*. (1). *ABHD5*, 1-acylglycerol-3-phosphate O-acyltransferase, also known as comparative gene identification-58 (CGI-58); C, carbon number; *CERS3*, ceramide synthase 3; *CYP4F22*, cytochrome P450, family 4, subfamily F, polypeptide 22; ELOV, elongation of very-long-chain fatty acid protein; *PNPLA1*, patatin-like phospholipase domain-containing 1; TG, triglyceride; ω, omega.

The biosynthesis of omega-O-acyl ceramides from fatty acid to acyl ceramide requires multiple steps (Fig. 1B). Several of these steps are associated with genetic variants in humans and cause disease when mutated. Whereas *ELOVL1* and *ELOVL4* variants, which are responsible for elongation to ultra-long fatty acids, cause both skin (scaling) and neurologic abnormalities (paraplegia, ataxia, brain atrophy), *CYP4F22*, *CERS3*, and *PNPLA1* (patatin-like phospholipase domain-containing 1) variants result in skin disease only, that is, excessive scaling of the skin. Interestingly, abnormalities in the most distal step of the pathway (Fig. 1B) can either be caused by *PNPLA1* variant or *ABHD5* variant and are associated with a similar scaly skin phenotype. All these entities are characterized by deficiency in omega-O-acyl ceramides and impaired permeability barrier function (2, 3).

Abnormal barrier function is a feature of genodermatoses, termed epidermal differentiation disorders (EDD) (4). This group of diseases exhibits thickened, scaly skin, often accompanied by inflammation, due to erroneous keratinocyte differentiation. Physiologically, epidermal differentiation results in the coordinated formation of a stratum corneum, the outermost skin layer. Essential components of this barrier are *i*) scaffolding structures; that is, the cornified envelope, surrounded by *ii*) highly organized lipid lamellae consisting of cholesterol, sphingomyelin/ceramide, and free fatty acids (5), including a critical complement of tissue-specific, very long-chain *N*-acyl fatty acids (6). An optimal composition of skin lipids prevents the ingress of harmful external substances and at the same time blocks uncontrolled fluid loss to the outside. If omega-O-acyl ceramide is not generated in sufficient quantities and positioned in the correct location, its ability to form a protein-bound ultra-long-chain-ceramide coating on the external surface of the cornified envelope leads to abnormal stratum corneum lipid composition and structure and impaired permeability barrier function (7, 8).

Subjects with *ABHD5*-syndromic (s)EDD or Chanarin-Dorfman syndrome (9), in contrast to those with *PNPLA1* variants, additionally exhibit pathologies in other organs, due to a lack of breakdown of triacylglycerol to free fatty acids, resulting in the accumulation of lipid droplets in specific organs. The organ involvement defines the disease as syndromic (hearing abnormalities and increased liver and muscle function parameters) (4), correlating with the role of *ABHD5* in lipid metabolism.

Schratter *et al*. (1) tested the effects of various naturally occurring missense mutations in *ABHD5* by combining coimmunoprecipitation with solid-phase interaction assays and immunofluorescence live-cell imaging. The authors demonstrate that the proximity of *ABHD5* with lipid droplets, specifically perilipins, and simultaneous enzymatic activation of *PNPLA1* is critical (Fig. 1C). This work demonstrates that *ABHD5* has multiple functions. It modulates the spatial proximity to lipid droplets, and it serves as a cofactor for *PNPLA1*.

Table 1 by Schratter *et al*. (1) illustrates the variability of the disease phenotype with different *ABHD5* variants. Genetic alterations affecting different parts of the 3-dimensional molecular structure of *ABHD5* have differential effects, which explain phenotype variability between subjects with *ABHD5*-sEDD. Some individuals have more severe liver involvement, whereas others have predominant skin involvement. A recent report even described a variant that causes skin disease only, with no other organ involvement (10). Schratter *et al*. (1) suggested that this is likely due to different molecular surfaces being involved in mediating the local positioning of *ABHD5* in different ways. Alternatively, multiple *PNPLA* enzymes, for example, *PNPLA3*, or other, hitherto unrecognized additional components of lipid droplet complexes may interact differently with *ABHD5* when it is genetically altered.

**Table 1:** Naturally occurring variants in genes relevant for omega-O-acyl ceramide synthesis

Lipid enzyme	Function	Nonsyndromic	Syndromic	Disease phenotype
ELOVL fatty acid elongase 1	Synthesis of C26 fatty acids and sphingolipids	No	Yes	pEDD, migratory erythematous scaling of the skin, spastic paraplegia, and dysmorphic facies
ELOVL fatty acid elongase 4	Synthesis of very-long-chain polyunsaturated fatty acids	No	Yes	Erythrokeratodermatous plaques, spastic paraplegia, developmental delay, and sometimes brain atrophy
*CYP4F22*	Catalyzes ULCFA ω-hydroxylation	Yes	No	Skin thickening and generalized fine scaling, can spare the trunk, sometimes self-improving
*CERS3*	Catalyzes the formation of ceramides from sphingoid base and acyl-coA substrates	Yes	No	Erythema, generalized fine scaling
*PNPLA1*	Transacylase transferring linoleic acid from triglycerides to omega-hydroxyceramides	Yes	No	Erythema, fine scales on flexor and extensor surfaces and facial skin, formerly known as erythrokertodermia variabilis et progressive
*ABHD5*	Interactions with members of the perilipin (PLIN) and patatin-like phospholipase domain-containing protein (*PNPLA*) protein families enhancing linoleic acid transesterification	Yes	Yes	Erythema, generalized fine scaling, ataxia, myopathy, hearing loss, and alopecia

*ABHD5*, 1-acylglycerol-3-phosphate O-acyltransferase, also known as comparative gene identification-58 (CGI-58); C26, carbon number 26; *CERS3*, ceramide synthase 3; *CYP4F22*, cytochrome P450, family 4, subfamily F, polypeptide 22; LOV, elongation of very-long-chain fatty acid protein; pEDD, palmoplantar epidermal differentiation disorder; *PNPLA1*, patatin-like phospholipase domain-containing 1; ULCFA, ultra-long-chain fatty acid.

By studying a rare disease, this work makes a valuable contribution to the understanding of EDD pathogenesis and the biochemistry of omega-O-acyl ceramide synthesis. The results reported by Schratter *et al*. should motivate the study of additional naturally occurring genetic variants relevant for the various steps in the pathway to further elucidate the biochemistry of ceramide synthesis. A continued search for mechanisms of disease pathogenesis in these disorders will provide us with a better understanding of the pathways and potential new therapeutic approaches.

While one logical approach to treating *ABHD5* deficiency is to apply the missing lipids exogenously, that is, acyl ceramide, the present work suggests that pharmaceutical compounds modulating the spatial positioning of *ABHD5* in relation to *PNPLA1* in the vicinity of lipid droplets may be another therapeutic option. Finally, gene transfer or gene editing would be promising, but in syndromic disease, the intervention needs to take place at early developmental stages. In summary, a better understanding of lipid droplet formation and composition in EDD may improve our ability to treat these disorders (11).

## Conflict of interest

The authors declare that they have no conflicts of interest with the contents of this article.
